# Cytosolic sensor STING in mucosal immunity: a master regulator of gut inflammation and carcinogenesis

**DOI:** 10.1186/s13046-021-01850-9

**Published:** 2021-01-23

**Authors:** Qiongyuan Hu, Quan Zhou, Xuefeng Xia, Lihua Shao, Meng Wang, Xiaofeng Lu, Song Liu, Wenxian Guan

**Affiliations:** 1 Department of Gastrointestinal Surgery, Nanjing Drum Tower Hospital, the Affiliated Hospital of Nanjing University Medical School, Nanjing, China; 2 Medical School of Nanjing University, Nanjing, China

**Keywords:** STING, Mucosal barrier, Intestinal inflammation

## Abstract

The stimulator of interferon genes (STING) connects microbial cytosolic sensing with host cell effector functions. STING signaling plays a central role in cyclic dinucleotides (CDNs) and DNA sensing to induce secretion of interferons and pro-inflammatory mediators. Although activated STING signaling favors antimicrobial progress and facilitates mucosal would healing, its role in mucosal immunity and gut homeostasis is paradoxical, ranging from positive and negative effects within the gut. In our review, we summarize recent advance of STING signaling in gut homeostasis and inflammation, especially focusing on its molecular basis in mucosal immune response. Deep understanding of the regulatory mechanisms of intestinal STING pathway could promote clinical manipulation of this fundamental signaling as a promising immunomodulatory therapy.

## Background

Persistent exposure of intestinal mucosa to a variety of microorganisms and bacterial metabolism reflects the biological necessity for a multifaceted, integrated epithelial and immune cell-mediated regulatory system [1]. Disruption of intestinal homeostasis plays an important role in the development of systemic inflammatory response, leading to tissue inflammation and organ injury. Acute and chronic conditions such as inflammatory bowel disease (IBD), sepsis, and gastrointestinal (GI) cancer are associated with the imbalance of gut homeostasis [2]. Abbreviated activation of innate and adaptive immune response could potentially induce the development of severe inflammatory condition in gut. Stimulation by bacterial-derived pathogen-associated molecular patterns (PAMPs) and damage-associated molecular patterns (DAMPs) provoke intestinal pattern recognition receptors (PPRs), which are involved in the intestinal immune response and inflammation [3]. This research direction recently developed into a novel dimension with cytosolic surveillance systems. The adaptor protein stimulator of interferon genes (STING) is a vital milestone in sensing nucleotide research. STING connects microbial cytosolic sensing with host cell effector functions. Cyclic dinucleotides (CDNs) are important bacterial metabolism and while DNA is presented in most microorganisms, both of which could activate STING signaling. Besides, STING can recognize host self-DNA, including nuclear DNA (nDNA) and mitochondrial DNA (mtDNA), conferring on STING an important role in host immune response [4, 5].

STING discovered by Barber et al. at 2008, is an endoplasmic reticulum (ER) adaptor that regulates intracellular DNA-mediated, type I interferon-dependent innate immunity [6]. However, the related pathway remains unknown at that moment. In 2013, Chen’s group discovered a new cytosolic DNA sensor cyclic GMP-AMP (cGAMP) synthetase(cGAS), declaring the arrival of “cGAS-STING” era [7, 8]. Since then, numerous studies have investigated that the activation of STING was essential for host defenses against viral and bacterial infections as well as cancer. However, increasing evidences showed that the excessive activation of STING could contribute to various diseases, including autoimmune and inflammatory diseases. Particularly, recent studies demonstrated that STING signaling was associated with intestinal homeostasis, i.e.., STING signaling could be beneficial or detrimental to gut barrier in different scenarios. It is therefore necessary to balance the conservation and activation of STING signaling in response to microbial PAMPs and self-DAMPs in gut.

## Activation of STING signaling

STING is an ER-resident protein in various cell types, including epithelial and endothelial cells, macrophages and dendritic cells (DCs). Activated STING is associated with enhanced secretion of type I interferon (IFN) and inflammatory cytokines in response to PAMPs and DAMPs. In steady state, cytoplasmic DNA degrades quickly through nucleases or endolysosomal compartments. In pathological conditions, self or microbial DNA accumulates in the cytosol, which was recognized by cGAS and its second messenger cGAMP [9]. cGAMP could translocate into adjacent cells via volume regulated anion channels or gap junctions, resulting in the activation of STING in neighboring cells [10, 11].

**Fig. 1:**
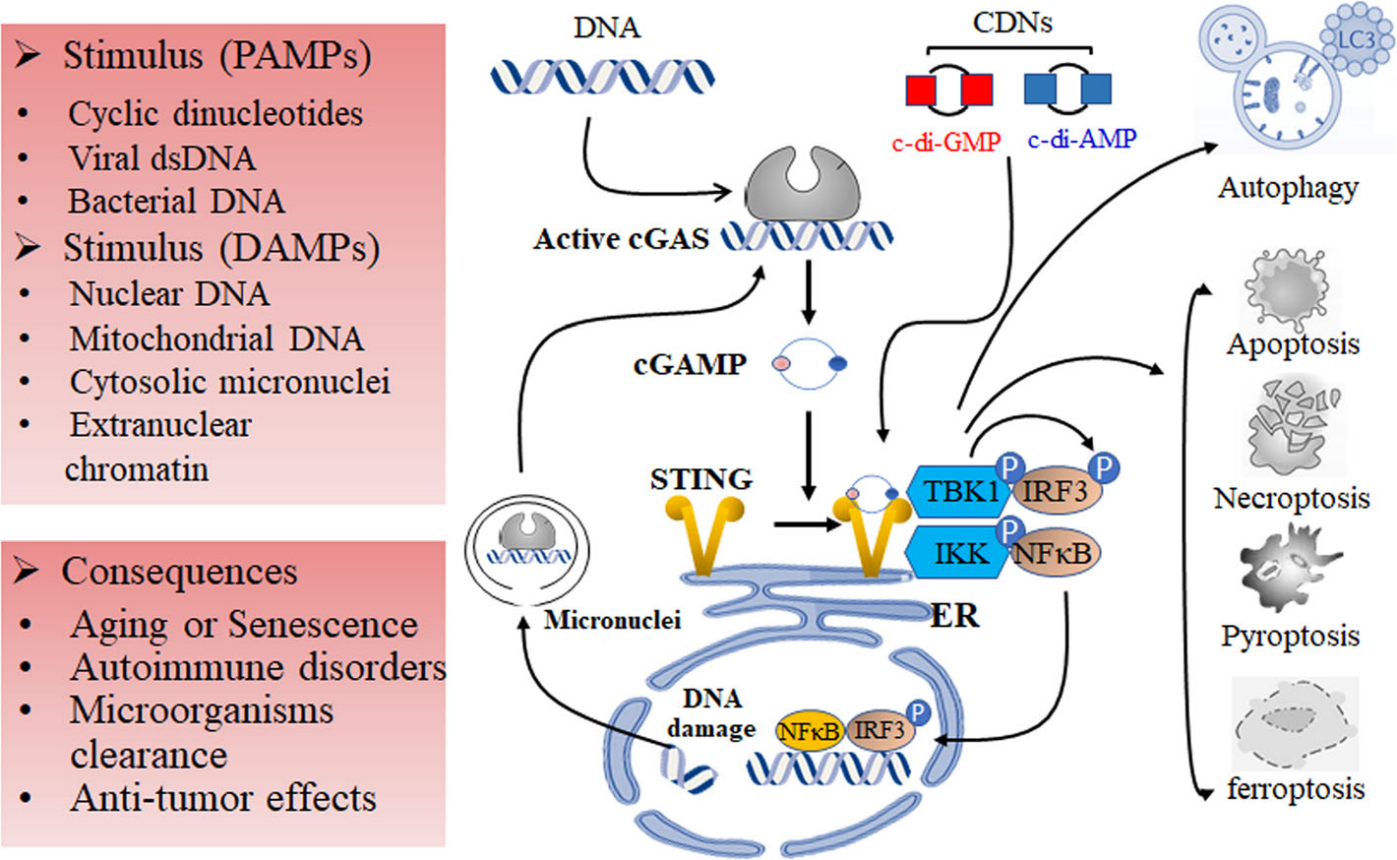
Overview of STING signaling. STING is activated by CDNs produced by bacteria or by cGAS following binding to cytosolic DNA. Activated STING in ER contributes to translocation of STING to Golgi, where interaction with TBK1 happens via the C-terminal tail of STING. Activated TBK1 can enable the recruitment and phosphorylation of IRF3 and NF-κB, which leads to enhanced production of type I interferon and inflammatory cytokines. STING signaling also participates in other cellular process, including autophagy and different types of cell death. CDNs, cyclic dinucleotides; cGAS, cyclic GMP–AMP synthase; ER, endoplasmic reticulum; TBK1, TANK-binding kinase 1; IRF3, interferon regulatory factor 3; NF-κB, nuclear factor-κB

Upon binding to cGAMP, STING traffics via Golgi apparatus and ER–Golgi intermediate compartment (ERGIC), regulated by the cytoplasmic coat protein complex II and ADP-​ribosylation factor (ARF) GTPases [12]. Activated STING subsequently recruits TANK- binding kinase 1 (TBK1), and phosphorylates interferon regulatory factor 3 (IRF3) and nuclear factor-κB (NF-κB). These transcriptional factors are capable to translocate from cytoplasm into nucleus to induce innate immune genes transcription, contributing to the production of type I IFN and inflammatory cytokines (Fig. 1).

STING signaling also plays an important role in autophagy, cell death, and senescence [13, 14]. Recently, Li et al. [15] found that mtDNA-STING pathway could induce autophagy-dependent ferroptotic cell death via lipid peroxidation. Intense efforts are underway to develop both inhibitors and activators of STING, which could be beneficial for the management of autoimmune disease and cancer [16, 17]. Pan et al. [18] identified MSA-2 as a potent STING agonist, and oral MSA-2 exhibited durable STING-dependent antitumor effect.

## Regulation and modification of STING signaling

Sophisticated regulation of STING signaling is critical to avoid excessive immune response. MicroRNAs were recently found to regulate STING expression. miR-210 [19], miR24-3p [20] and MiR-576-3p [21] could inhibit STING expression at both translational and protein levels. In contrast, miR29a and miR378b could activate STING signaling [22]. In resting cells, STING bounds to the Ca^2+^ sensor stromal interaction molecule 1 (STIM1) within ER. Once STING binds to the cGAMP, the interaction between STING and ER-resident protein STIM1 was disrupted, initiating STING translocation from ER to Golgi [23]. How STING dimerizes and trafficks from ER to ERGIC has been explored recently. ER-associated protein ZDHHC1 is important for innate immune response, and overexpression of ZDHHC1 activated the promoter of INF-β. ZDHHC1 was colocalized with STING in ER, and was constitutively associated with the dimerization of STING and recruitment of TBK1 [24].

Post-translational modifications (PTMs) of STING have been extensively investigated recently. S358, Ser366, and Y245 are three phosphorylation sites for STING activation (Table 1) [25, 26]. Conversely, autophagy-related gene (ULK1) induces S366 phosphorylation, contributing to STING degradation and abolishment of IFN and inflammatory cytokines production [27]. The tyrosine-protein phosphatase nonreceptor type 1 and 2, and PPM1A could dephosphorylate STING, respectively [28, 29], leading to degradation of STING.

**Table 1:** Post-translational modifications (PTMs) of STING protein

Type of PTMs	Sites	Enzyme	Consequences	Ref
**STING protein**				
Phosphorylation	Y245	SRC	dimerization and stability of STING	26
	S358	TBK1	Recruitment of TBK1	27
	S366	TBK1	Recruitment of IRF3	27
	S366	ULK1	Induces degradation of STING	28
Dephosphorylation	Y245	PTPN1/2	Degradation by a proteasome pathway	29
	S358	ULK1	Suppression of STING activity	30
Ubiquitination				
K11-linked polyubiquitination	K150	RNF26	Promotes stability of STING	31
K27-linked polyubiquitination	K137/150/ 224/236	AMFR	Promotes recruitment of SITNG	32
K63-linked polyubiquitination	K20/150/ 224/236	TRIM32	promotes the interaction of STING with TBK1	33
K63-linked polyubiquitination	K224/236/ 289/338	MUL1	Enhances trafficking of STING	34
K63-linked polyubiquitination	K150	TRIM56	Recruitment of TBK1 and induction of INF-β	35
K48-linked polyubiquitination	K275	TRIM30α	Proteasomal degradation of STING	36
K48-linked polyubiquitination	K150	RNF5	Proteasomal degradation of STING	37
Palmitoylation	C88/91	DHHC	Enhances trafficking of STING and type I IFN response	38
Nitro- alkylation	C88/91	N.D.	Inhibits normal palmitoylation process	39
Carbonylation	C88	GPX4	inhibition of STING trafficking from the ER to the Golgi	40
Sumoylation	K338	TRIM38	Promotes oligomerization and stability of STING	41
Desumoylation	K388	SENP2	Lysosomal degradation of STING	41
Oxidation	C147	ROS	Inhibition of STING polymerization	42

Besides phosphorylation, other types of PTMs including polyubiquitination, palmitoylation, nitro-alkylation and sumoylation were observed as well. Several types of polyubiquitin modifications such as K11-, K27-, K63- and K48-linked polyubiquitination regulate STING expression level and activity in both steady and stimulated cells. K11-, K27-, K63-associated polyubiquitination was crucial for stabilizing STING and recruiting TBK1 [30–34]; while K48-linked polyubiquitination was associated with STING degradation in a proteasome pathway [35, 36].

Trafficking of STING from ER to Golgi apparatus is essential for the activation of downstream pathway. STING is palmitoylated at Cys88 and Cys91 at the Golgi apparatus [37]. Treatment of nitro-fatty acids could modifies Cys88 and Cys91 of STING through nitro-alkylation in ER, inhibiting normal palmitoylation [38]. Additionally, STING was recently showed to be carbonylated by lipid peroxidation, and STING carbonylation inhibited STING palmitoylation and subsequent activation [39]. Sumoylation plays an important role in protein stability. Hu et al. [40] showed that sumoylated STING following DNA stimulation promoted oligomerization and prevented its degradation [40]. Additionally, Reese’s group recently demonstrated that reactive oxygen species could suppress IFN response by oxidizing STING, indicating that redox modification also plays an important role in STING modification [41].

## STING signaling in gut homeostasis

Persistent exposure of intestinal mucosa to tremendous microorganisms and their metabolites reflects the biological necessity for a multilevel, integrated epithelial and immune cell-mediated regulatory system. Impaired mucosal barrier and disruption of intestinal homeostasis lead to bacterial translocation and mucosal inflammatory response. Recent studies indicated that PAMPs and DAMPs are involved in intestinal STING activation that help to shape gut homeostasis.

### STING signaling in enteropathogenic bacterial infections

Infectious diarrhea caused by enteropathogenic bacteria is a major cause of morbidity and mortality worldwide [42]. Innate immunity plays a crucial role in preventing enteropathogenic bacterial infections. STING signaling recently provided crucial insights into antimicrobial and immunomodulatory therapeutics against pathogen.

*Listeria monocytogenes* is a gram positive facultative intracellular bacterium, and type I IFN is essential for host defense against *Listeria* disease. Released c-di-AMP into host cytoplasm is dependent on multidrug efflux pumps (MDRs) that induces host cytosolic surveillance pathway in murine cells [43]. However, Hansen et al. [44] investigated that in human macrophages, *Listeria* DNA rather than c-di-AMP induces type I IFN response that depends on cGAS-STING pathway. Additionally, they reported that *Listeria* DNA-induced IFNβ expression is associated with bacteriolysis in human macrophage cytosol. Further studies are necessary to identify the interaction between innate and protective immunity following *Listeria* infection.

Unlike *Listeria*, *Shigella flexneri*, a causative agent of bacillary dysentery, was found to limit STING signaling. Two Shigella type 3 secretion system (T3SS) effector proteins (IpaJ and VirA) were capable to disrupt immune response [45]. Dobb et al. [46] found that *Shigella* IpaJ could suppress STING signaling and type I IFN response through inhibiting its translocation from ER to ERGIC. VirA could cause STING retention in ERGIC, but failed to inhibit type I IFN. Moreover, Dong et al. [47] found that inactivation of Rab1 by VirA caused increased bacterial burden in cytoplasm by suppressing autophagy-mediated immune response.

*Salmonella* is another intracellular pathogen that causes severe gastrointestinal and systemic infection. STING-deficient mice exhibited increased mortality rate compared to WT counterparts following oral administration of *Salmonella typhimurium* [48]. Park et al. [49] recently demonstrated that STING-IRF1-dependent signaling in DCs was able to drive TH17 polarization in response to evaded entero-pathogen (e.g., *Salmonella*) in gut.

### STING is associated with sepsis‐induced intestinal injury

Gut has been suggested as the ‘driver’ of sepsis and organ injury [50]. Gut epithelium, immune system and microbiome were significantly disrupted during sepsis [51]. Our recent study found that STING was remarkably activated in the gut of sepsis patients, which was associated with exacerbated historical injury and elevated intestinal epithelial cell apoptosis [52]. Our findings suggest a critical involvement of STING-induced excessive inflammation and intestinal epithelial cells (IECs) apoptosis sensing by CDNs and host DNA during sepsis, leading to intestinal barrier damage, increased intestinal permeability [52]. Zeng et al. [53] also demonstrated that STING depletion improved survival in both lethal endotoxemia and polymicrobial sepsis model, which was associated with leukocyte infiltration and tissue destruction.

STING was originally discovered as a cytosolic nucleic-acid sensor dependent on cGAS, a DNA-binding protein. Emerging evidence has demonstrated that circulating DNA derived from injured host cells or invading pathogens was significantly increased in sepsis patients, and was associated with adverse outcomes [54, 55]. However, unlike STING^−^/^−^, cGAS depletion failed to prevent against sepsis-induced mortality and tissue injury, implying that other DNA sensors may play a more important role during sepsis.

**Fig. 2:**
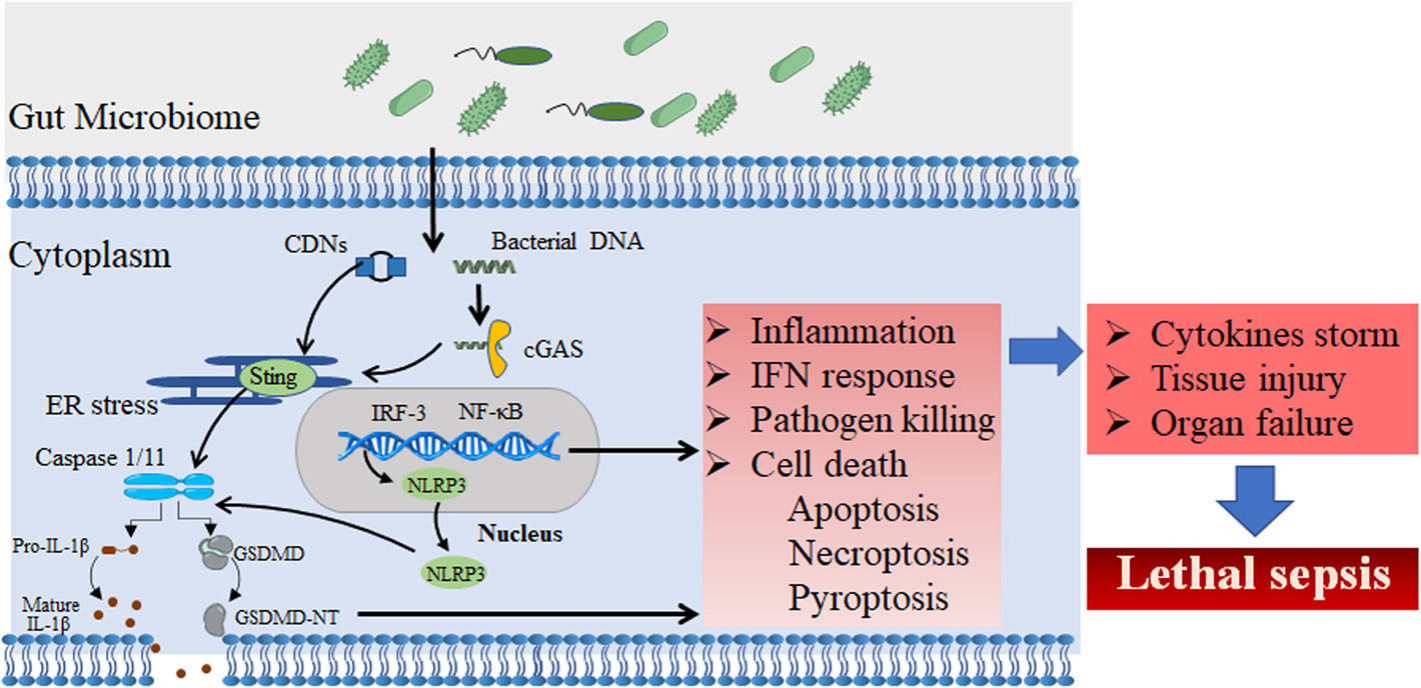
STING activates multiple pathways inducing sepsis progression. Microbes can induces STING signaling, which activate multiple pathways, contributing to inflammation, IFN response, and cell death

Zhang et al. [56] identify an alternative STING pathway in organ failure. STING-mediated GSDMD (a pore-forming protein) cleavage by caspase1/caspase11 or caspase8 induces tissue factor F3 release and lethal coagulation independent of classical downstream of STING signaling. Li et al. [57] recently investigated that STING leads to lipopolysaccharide-induced tissue dysfunction, inflammation, apoptosis and pyroptosis by activating NLRP3 (NOD-like receptor family, pyrin domain containing 3) signaling (Fig. 2). Therefore, both canonical and alternative STING signaling are involved in the development of sepsis. However, the specific mechanism that how STING is precisely activated in infected cells or septic gut remains largely unknown.

### STING signaling in IBD-associated intestinal disorder

STING has been suggested as a negative regulator in various autoinflammatory diseases, such as systemic lupus erythematosus, and rheumatoid arthritis [58]. However, the role and potential mechanism of STING signaling in IBD remain largely unknown.

IBD, including Crohn’s disease (CD) and ulcerative colitis, is a chronic and relapsing immune disorder. Recent studies suggested that DAMPs released from extensively inflamed mucosa act as a ‘motor’ in inducing and maintaining intestinal inflammation. DAMPs are responsible for mucosal inflammatory insults in both human and animal model. Our recent study discovered that mitochondrial DAMPs (mtDNA) released from gut mucosa could induce intestinal inflammatory response dependent on STING signaling, and the induction of STING signaling in intestine of active CD patients supports a potential pathogenic role of STING in IBD [3, 52].

Canesso et al. [48] observed that STING knockout mice showed a higher susceptibility to T-cell-induced and DSS-induced colitis compared to WT littermates, suggesting a protective effect of STING pathway in gut homeostasis. Barber’s group confirmed that STING-deficient mice were prone to polyp formation and intestinal inflammation in response to DSS stimulation [59]. They suggested that STING signaling was crucial for wound healing and antimicrobial processes by preventing microbes invading into lamina propria where they could induce excessive inflammatory response. These studies consistently demonstrated an association between STING deficiency and aggravated intestinal inflammation.

It is plausible that treatment of STING agonist can improve DSS-induced colitis. However, Martin et al. [60] recently demonstrated that STING agonist deteriorated DSS-induced colonic injury, significant weight loss and colonic shortening. Ahn et al. also found that STING deficiency could prevent colitis due to impaired IL-10 production [59]. IL-10 is an anti-inflammatory cytokine that requires the activation of transcription factors IRF3 and NFκB [61]. Therefore, it is reasonable that STING can induce the secretion of IL-10. Without STING-dependent IL-10 secretion, increased inflammatory mediators triggered by STING could induce a higher inflammatory state. Meanwhile, Aden et al. [62] demonstrated that activated STING signaling in intestinal epithelial cell induces a strong TNF and interferon-stimulated genes response, which leads to excessive ileal inflammation and widespread epithelial cell necroptosis.

In summary, STING signaling may play a paradoxical role in gut homeostasis during the development of IBD. STING signaling determines the outcomes of IBD depending on types of immune cells involved. Gut mucosal immune response is regulated by several innate receptors beyond STING. DSS-induced gut inflammation may enable microbes access to the lamina propria where they could activate STING-independent inflammatory signaling, such as Toll-like receptors and NOD-like receptors [3].

cGAS is a fundamental upstream protein of STING. Interestingly, cGAS may plays an different role compared to STING in the development of IBD. cGAS-deficient mice showed modest inflammatory response and polyp formation by DSS treatment [59], and cGAS inhibitor remarkably alleviated the clinical signs of colitis in mice [63]. It is still unclear why cGAS-deficient mice exhibited moderate intestinal inflammation following DSS challenge compared with STING-deficient mice. It is possible that microbiota-derived CDNs play a more crucial role in the development of colitis than microbial or self-free cytosolic DNA species. Therefore, further studies need to compare the pathogenic effect between bacteria-derived CDNs and genomic DNA in the development of colitis.

## STING signaling and cancer

### Anti‐tumorigenesis effect of STING

Recent advance has suggested an important role of STING signaling in the development of GI cancer. Increased tumor load was observed in STING knockout mice following azoxymethane/dextran sodium sulfate (AOM/DSS) treatment that can cause colitis-associated cancer (CAC). The deficiency of STING was associated with increased inflammatory response and significant dysplasia in colorectal tissues.

**Fig. 3:**
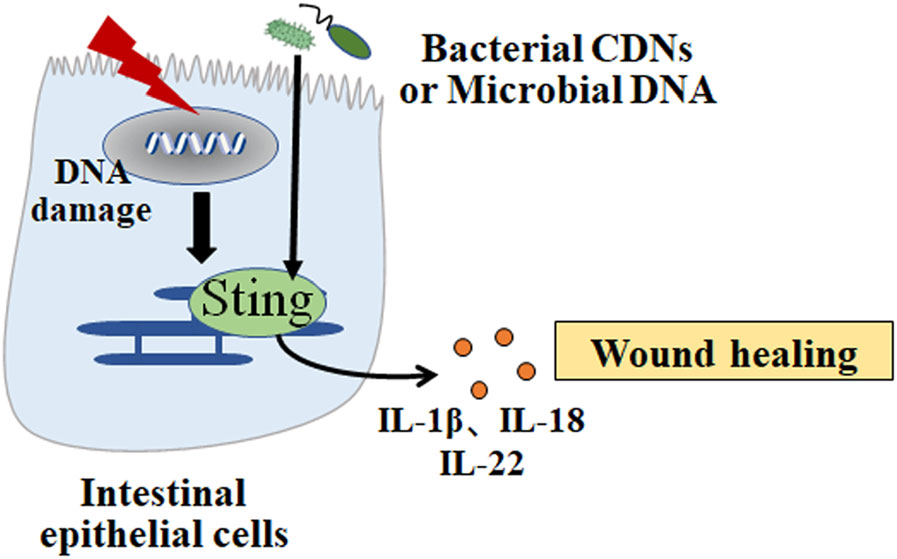
STING promotes would healing. Self and microbial DNA, and CDNs activates intrinsic STING pathway in intestinal cells, which enhance the production of IL-1β or IL-18 that promote intestinal would healing

STING-dependent signaling is capable to induce IL-1 and IL-18 production. Decreased expression of IL-1β and pre-caspase-1 were observed in colon tissues of STING knockout mice [59]. Consistently, IL-18 and IL-22BP expression were found decreased in the colon tumor of AOM/DSS-treated STING knockout mice compared to WT mice [64]. It is possible that cell repair factors (IL-1β, IL-18) activated by STING signaling promotes intestinal would healing, which can prevent microbiome translocating into lamina propria where they can activate inflammatory pathway (Fig. 3). When natural wound healing was interrupted, persistent inflammation would disrupt the intestinal microbial composition, contributing to further inflammatory response, DNA damage and cancer development [65, 66]. In tumors and cancer cell lines, STING signaling is frequently silenced or mutated, indicating its role in limiting tumor growth. Decreased STING expression was positively associated with tumor invasion depth, lymph mode metastasis, and reduced patients’ survival in gastric cancer patients [67]. Nevertheless, no mutation was showed in genome encoding cGAS-STING signaling through gene sequence analysis and the suppression may be attributed to epigenetic modification, such as hypermethylation. Collectively, suppression of STING function is a crucial obligation for tumorigenic process.

**Fig. 4:**
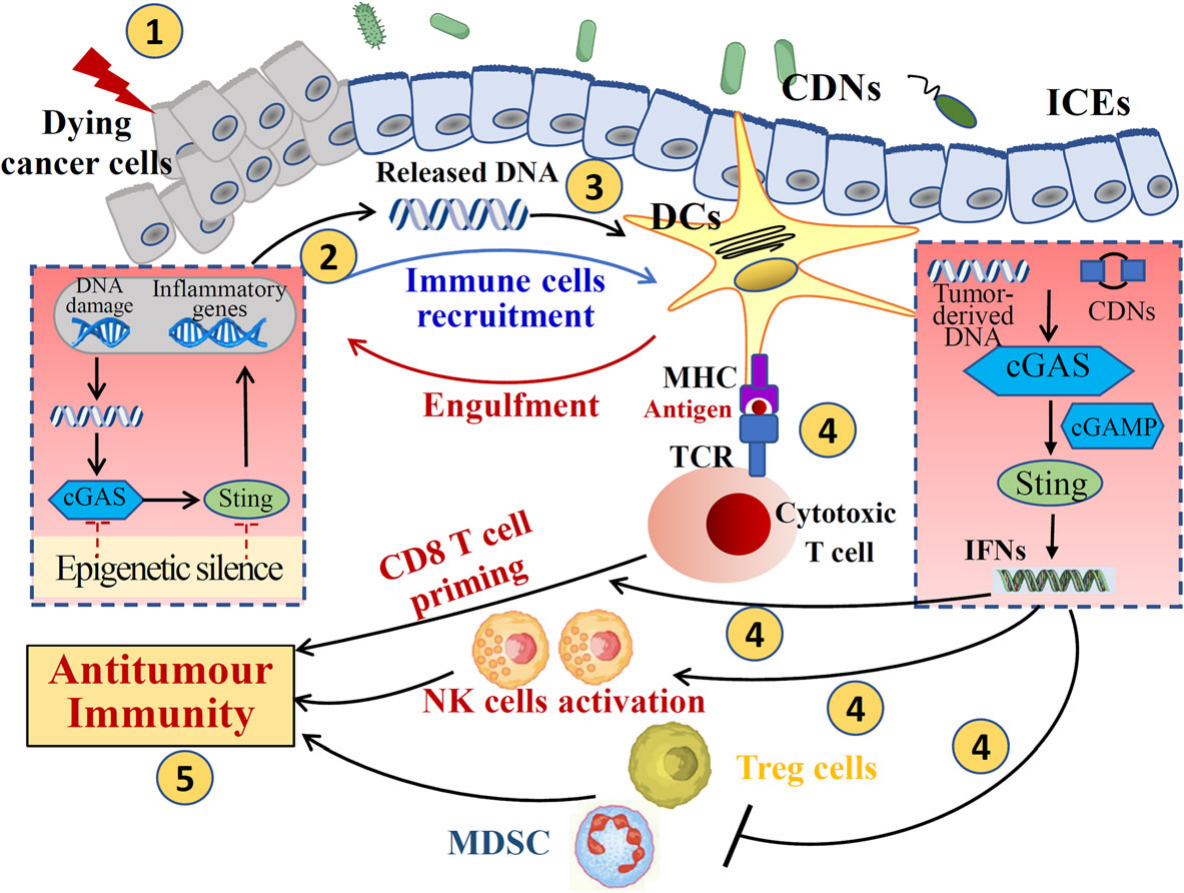
STING-dependent antitumor effect in gastrointestinal cancer. Dying tumor cells (1) could release a large amount of DNA (2); tumor-derived DNA are engulfed by DCs, and activates STING-dependent type I IFN response in the phagocyte (3), which facilitates cross-presentation and antitumor CD8^+^ T responses (4). Type I IFNs was also showed to suppress the function of immune suppressive cell such as Tregs cells and MDSCs, and induce the activation of NK cell (4), which play an important role in the destruction of tumor cells and antitumor immunity (5). DCs, dendritic cells

Adaptive T cell response is fundamental for controlling and eradicating tumor cells. DCs and IFN are crucial to induce adaptive T cell response [68]. It is suggested that DCs can engulf necrotic tumors cell, and the tumor-derived DNA activates STING signaling and enhances the production of type I IFN in DCs [69], which acts in an autocrine or paracrine pattern and activates the generation of additional proteins within the DCs to induce cross-presentation and T cell activation. Early Colorectal cancer patients showed higher STING expression with increased intratumoral CD8^+^ T cell infiltration and less frequent lymphovascular invasion. Moreover, intratumoral STING treatment was suggested to inhibit colon cancer progression through enhanced CD8^+^ T cells [70]. Other PRRs, including TLR and RIG-like receptors (RLRs), also activate type I IFN response. However, Woo et al. [71] showed that STING-IRF3 pathway-dependent type I IFN, rather than other IFN-associated signaling, in DCs is required for endogenous antitumor CD8^+^ T cell response. Tumor-derived DNA captured by cGAS in DCs as well as tumor cells themselves in tumor microenvironment (TME) is the primary driving force to activate STING signaling to induce the production of type I IFN and the tumor-specific CD8^+^ T cell priming (Fig. 4). Additionally, recent studies also suggested that STING activation in TME can suppress the function of immunosuppressive cells (regulatory T cells and MDSCs), and induce the activation of NK cells, which could promote destruction of tumor cells [72]. The important role of STING signaling in triggering anti-tumor T responses has inspired interests in the development of STING agonists for cancer therapy. Several studies suggested that CDNs that bind human STING exhibited antitumor effect in animal studies [73]. STING agonists were suggested to be effective against tumors that were resistant to programmed cell death protein 1 (PDL1) blockade [74]. Chin et al.[75] recently identified a non-nucleotide, small-molecule STING agonist, termed SR-717, that promoted the activation of CD8^+^ T, NK cells, and DCs in; and facilitated antigen cross-priming. Additionally, SR-717 also induced the expression PD-L1 in a STING dependent manner [75].

### The protumor effect of STING signaling

Recent evidence suggested that STING pathway may play an important role in malignant transformation mainly by activating immune suppressive tumor microenvironment and inducing tumor metastasis. Immune suppressive landscape is indicated during chronic activation of STING pathway. The immune checkpoint indoleamine 2,3-dioxygenase (IDO) is marker of immune suppressive TME. Decreased IDO was detected in STING-KO mice in TME of lung cancer model [76]. Activation of STING signaling is also associated with increased expression of CCR2 in colon cancer [77]. Elevated expression of CCR2 in MDSCs induced the aggregation of tumor-promoting monocytes. In addition, increasing evidence demonstrated that activated STING in T cells could damage adaptive immune system and accelerate tumorigenesis [78]. However, potential mechanisms of STING in immune suppressive environment remains largely unknown, further studies are needed to identify the relationship between STING and TME.

STING was recently suggested to induce tumor metastasis. Activation of STING signaling was associated with increased inflammatory response and upregulation of NFκB pathway, which contribute to epithelial-to-mesenchymal transition and metastasis [79]. Chen et al. [80] demonstrated that STING was activated in astrocytes and subsequently induced secretion of inflammatory cytokines, leading to brain metastasis of lung cancer and breast cancer. Potential relationship between STING and cancer metastasis remains unknown. One possible mechanisms may be related to the degree of STING levels. Gulen et al. [81] recently indicated that magnitude of STING signaling determined the activation of apoptotic programs in macrophages and T lymphocytes, indicating that regulation of STING levels was associated with distinct downstream effector programs. Our recent studies also showed that degree of STING activation is associated with disease outcomes [82]. Further investigation was needed to uncover the molecular context by which activated STING facilitates tumor metastasis.

### The diet, gut microbiota, and STING signaling

The contribution of diet to regulating the microbiota and its important role in orchestrating the host–microbiota crosstalk has been well-recognized [83]. Nutrients can directly interact with microorganisms to promote or inhibit their growth, and dietary interventions could trigger structural and functional alterations in the gut bacteria [83]. To our knowledge, There are no studies to investigate the effects of diet on the intestinal mucosal barrier via regulating STING signaling. STING play an important role in regulating the composition of gut microbe. Canesso et al. [48] showed that significant differences in relative abundance of bacteria populations between STING-/- and WT mice. There was a greater fecal output in the Proteobacteria and a reduction in the Actinobacteria phylum in the feces from STING-/- mice. Additionally, recent evidence suggests that high-fat diet is associated with activation of STING signaling. Therefore, we consider that diet may modulate gut microbiota through regulating STING signaling, and further studies are need to clarify the potential mechanisms.

### Delivery systems of STING agonist

STING agonists have also been suggested to be experimentally useful as adjuvants in anticancer vaccine studies [69]. However, excessive expression of STING in T lymphocytes contribute to T-cell apoptosis, a phenomenon that appeared cell specific as DCs or macrophages did not show such sensitivity [84]. Hence, combination of STING agonists and effective adjuvant/antigen delivery system plays an important role in cancer vaccines, which could specifically target immune cells, including DCs, macrophages and NK cells [85]. Liposomes, polymers, and hydrogels have been suggested efficiently to deliver STING agonist. Koshy et al. [86] investigated that cationic liposomes with polyethylene glycol were used to encapsulate cGAMP to facilitate its cytosolic delivery, leading to antitumor activity. Moreover, Cheng et al. [87] demonstrated that liposomal nanoparticle-delivered cGAMP directed both mouse and human macrophages; increased MHC and costimulatory molecule expression; and enhance T cell infiltration.

Multifunctional polymers are also effective tools to deliver STING agonist. Gao’s group reported a synthetic polymeric nanoparticle, PC7A nanoparticles, which generated a strong cytotoxic T-cell response dependent on STING signaling [88]. Similarly, Zhou et al. developed an engineering polymeric prodrug nanoplatform for vaccination immunotherapy of cancer, which dramatically promoted DC maturation through activating STING signaling [89]. Recently, Lu et al. [90] developed PLGA microparticles for long-term, pulsatile release of STING agonist for cancer immunotherapy. Moreover, other polymer (dextran microparticles) was suggested to transport STING agonist and realize antitumor effect [91, 92].

Different from liposomes and polymer nanoparticles, the advantages of hydrogels as carriers are the local and controlled release of STING agonist, leading to recruitment and activation of immune cells. A recent study developed a novel biomaterial called ‘STINGel’, which was an injectable peptide hydrogel that localized and provided control release of CDN delivery [93]. STINGel improves survival in a challenging murine oral cancer model. Additionally, Cui’s group recently designed a self-assemble hydrogel that can locally deliver STING agonists to activate DCs and NK cells, contributing to long-term immune memory and systemic immune surveillance, thereby reducing tumor immunosuppression and enhancing the efficacy of a wide range of cancer therapies [94]. However, none of these delivery systems have been used in clinic. Additionally, excessive activation of STING signaling could induce systemic inflammation and a cytokine storm. Thus, greater sight into the mechanism could drive us to develop more specific agonist and achieved safe, personalized, and valid therapies.

## Type I IFN in autoimmune diseases and cancer

Type I IFN (mainly IFN-α and IFN-β) are the main effectors of STING pathway-dependent modulation of innate immunity. Type I IFN pathway is broadly implicated in autoimmune diseases and cancer [95]. Type I IFN is activated in patients with several systemic autoimmune diseases, which seems to be of major importance in the disease process. Both of IFN-α and IFN-β could contribute to systemic lupus erythematosus (SLE) pathogenesis, and blockade of IFNAR provides effective therapy for systemic autoimmune disease [96]. Moreover, type I IFN are increasingly recognized for their role in regulating anti-tumour immune responses. IFN-α/β can directly target tumor cells through inducing apoptosis and growth arrest [97]. Additionally, Targeting type I IFN to tumor microenvironment promotes anti-tumor activity through host adaptive immunity that is T-cell dependent [97]. Several studies demonstrated that the intra-tumoural expression levels of type I IFN or of IFN-stimulated genes correlate with favourable disease outcome in several cohorts of cancer patients [98]. Therefore, therapies designed to increase the intra-tumoural concentration of type I IFN can have antineoplastic effects following the induction of anticancer immune responses. Although type I IFN signaling is required to trigger anti-tumor immunity, emerging evidence indicates that chronic activation of type I IFN pathway may be involved in mediating resistance to different cancer treatments [98]. Therefore, Strategies able to temporarily block IFN-signaling, preferably in cancer cells only, could be useful to limit chronic exposure to IFN and restore responsiveness to treatment.

## Conclusions

It has been clear that STING plays an important role in intestinal mucosal immunity. The effect and mechanism of STING signaling varies depending on different scenarios. Emerging evidence has showed that activation of STING signaling enhance anti-cancer immune response, and STING agonists have been suggested as promising anti-tumor therapy, including target therapy and immunotherapy. It is worth noting that STING as a vital modulation of inflammation and IFN response, could instigate tumor development and metastasis. Additionally, degree of STING molecule is associated with cancer outcome. Therefore, the tumor status and therapeutic windows should be carefully evaluated before using STING agonists or antagonists. Further studies are expected to help clinician select appropriate STING modulators according to specific condition.

## Data Availability

There are no new data associated with this review article.

## Abbreviations

STING: Stimulator of interferon genes
CDNs: Cyclic dinucleotides
IBD: Inflammatory bowel disease
GI: Gastrointestinal
PAMPs: Pathogen-associated molecular patterns
DAMPs: Damage-associated molecular patterns
PRRs: Pattern recognition receptors
mtDNA: Mitochondrial DNA
ER: Endoplasmic reticulum
DCs: Dendritic cells
IRF3: Interferon regulatory factor 3
NF-κB: Nuclear factor-κB
PTMs: Post-translational modifications
CAC: Colitis-associated cancer
PDL1: Programmed cell death protein 1

## References

[1] de Jong PR, Gonzalez-Navajas JM, Jansen NJG. The digestive tract as the origin of systemic inflammation. Critical Care 2016, 20. 10.1186/s13054-016-1458-3 PMC5067918 27751165

[2] Kurashima Y, Goto Y, Kiyono H (2013). Mucosal innate immune cells regulate both gut homeostasis and intestinal inflammation. Eur J Immunol.

[3] Hu Q, Ren Y, Slade DA, Zhou Q, Wu X, Huang J, Gu G, Wang G, Ren J, Li J (2019). Damps’ role in inflammatory bowel disease: a paradoxical player of mtDNA-STING signaling pathway in gut homeostasis. Sci Bull.

[4] Ahn J, Barber GN (2014). Self-DNA, STING-dependent signaling and the origins of autoinflammatory disease. Curr Opin Immunol.

[5] Ahn J, Ruiz P, Barber GN (2014). Intrinsic Self-DNA Triggers Inflammatory Disease Dependent on STING. J Immunol.

[6] Ishikawa H, Barber GN (2008). STING is an endoplasmic reticulum adaptor that facilitates innate immune signalling. Nature.

[7] Gao DX, Wu JX, Wu YT, Du FH, Aroh C, Yan N, Sun LJ, Chen ZJJ (2013). Cyclic GMP-AMP Synthase Is an Innate Immune Sensor of HIV and Other Retroviruses. Science.

[8] Li XD, Wu JX, Gao DX, Wang H, Sun LJ, Chen ZJJ (2013). Pivotal Roles of cGAS-cGAMP Signaling in Antiviral Defense and Immune Adjuvant Effects. Science.

[9] Ahn J, Barber GN. STING signaling and host defense against microbial infection. Experimental and Molecular Medicine 2019, 51. 10.1038/s12276-019-0333-0 PMC6906460 31827069

[10] Ablasser A, Schmid-Burgk JL, Hemmerling I, Horvath GL, Schmidt T, Latz E, Hornung V (2013). Cell intrinsic immunity spreads to bystander cells via the intercellular transfer of cGAMP. Nature.

[11] Zhou C, Chen X, Planells-Cases R, Chu J, Wang L, Cao L, Li Z, Lopez-Cayuqueo KI, Xie Y, Ye S (2020). Transfer of cGAMP into Bystander Cells via LRRC8 Volume-Regulated Anion Channels Augments STING-Mediated Interferon Responses and Anti-viral Immunity. Immunity.

[12] Yang Q, Shu H-B (2020). Deciphering the pathways to antiviral innate immunity and inflammation. Advances in immunology.

[13] Hu Q, Knight PH, Ren Y, Ren H, Zheng J, Wu X, Ren J, Sawyer RG. The emerging role of stimulator of interferons genes signaling in sepsis: Inflammation, autophagy, and cell death. Acta Physiologica 2019, 225(3). 10.1111/apha.13194 30269441

[14] Li T, Chen ZJ (2018). The cGAS-cGAMP-STING pathway connects DNA damage to inflammation, senescence, and cancer. J Exp Med.

[15] Li C, Zhang Y, Liu J, Kang R, Klionsky DJ, Tang D. Mitochondrial DNA stress triggers autophagy-dependent ferroptotic death. Autophagy 2020. 10.1080/15548627.2020.1739447 PMC8078708 32186434

[16] Hopfner K-P, Hornung V. Molecular mechanisms and cellular functions of cGAS-STING signalling. Nature Reviews Molecular Cell Biology 2020. 10.1038/s41580-020-0244-x 32424334

[17] Chen Q, Sun L, Chen ZJ (2016). Regulation and function of the cGAS-STING pathway of cytosolic DNA sensing. Nat Immunol.

[18] Pan BS, Perera SA, Piesvaux JA, Presland JP, Schroeder GK, Cumming JN, Trotter BW, Altman MD, Buevich AV, Cash B, et al: An orally available non-nucleotide STING agonist with antitumor activity. Science 2020, 369(6506). 10.1126/science.aba6098 32820094

[19] Xu T, Chu Q, Cui J (2018). Rhabdovirus-Inducible MicroRNA-210 Modulates Antiviral Innate Immune Response via Targeting STING/MITA in Fish. J Immunol.

[20] Shen A, Zheng D, Luo Y, Mou T, Chen Q, Huang Z, Wu Z (2020). MicroRNA-24-3p alleviates hepatic ischemia and reperfusion injury in mice through the repression of STING signaling. Biochem Biophys Res Commun.

[21] Yarbrough ML, Zhang K, Sakthivel R, Forst CV, Posner BA, Barber GN, White MA, Fontoura BMA. Primate-specific miR-576-3p sets host defense signalling threshold. Nature Communications 2014, 5. 10.1038/ncomms5963 PMC4170571 25232931

[22] Shah AU, Cao Y, Siddique N, Lin J, Yang Q. miR29a and miR378b Influence CpG-Stimulated Dendritic Cells and Regulate cGAS/STING Pathway. Vaccines 2019, 7(4). 10.3390/vaccines7040197 PMC6963666 31779082

[23] Srikanth S, Woo JS, Wu B, El-Sherbiny YM, Leung J, Chupradit K, Rice L, Seo GJ, Calmettes G, Ramakrishna C (2019). The Ca2 + sensor STIM1 regulates the type I interferon response by retaining the signaling adaptor STING at the endoplasmic reticulum. Nat Immunol.

[24] Zhou Q, Lin H, Wang S, Wang S, Ran Y, Liu Y, Ye W, Xiong X, Zhong B, Shu H-B (2014). The ER-Associated Protein ZDHHC1 Is a Positive Regulator of DNA Virus-Triggered, MITA/STING-Dependent Innate Immune Signaling. Cell Host Microbe.

[25] Liu S, Cai X, Wu J, Cong Q, Chen X, Li T, Du F, Ren J, Wu Y-T, Grishin NV (2015). Phosphorylation of innate immune adaptor proteins MAVS, STING, and TRIF induces IRF3 activation. Science.

[26] Zhong B, Yang Y, Li S, Wang Y-Y, Li Y, Diao F, Lei C, He X, Zhang L, Tien P (2008). The Adaptor Protein MITA Links Virus-Sensing Receptors to IRF3 Transcription Factor Activation. Immunity.

[27] Konno H, Konno K, Barber GN (2013). Cyclic Dinucleotides Trigger ULK1 (ATG1) Phosphorylation of STING to Prevent Sustained Innate Immune Signaling. Cell.

[28] Xia T, Yi X-M, Wu X, Shang J, Shu H-B (2019). PTPN1/2-mediated dephosphorylation of MITA/STING promotes its 20S proteasomal degradation and attenuates innate antiviral response. Proc Natl Acad Sci USA.

[29] Li Z, Liu G, Sun L, Teng Y, Guo X, Jia J, Sha J, Yang X, Chen D, Sun Q. PPM1A Regulates Antiviral Signaling by Antagonizing TBK1-Mediated STING Phosphorylation and Aggregation. Plos Pathogens 2015, 11(3). 10.1371/journal.ppat.1004783 PMC4376777 25815785

[30] Qin Y, Zhou M-T, Hu M-M, Hu Y-H, Zhang J, Guo L, Zhong B, Shu H-B. RNF26 Temporally Regulates Virus-Triggered Type I Interferon Induction by Two Distinct Mechanisms. Plos Pathogens 2014, 10(9). 10.1371/journal.ppat.1004358 PMC4177927 25254379

[31] Wang Q, Liu X, Cui Y, Tang Y, Chen W, Li S, Yu H, Pan Y, Wang C (2014). The E3 Ubiquitin Ligase AMFR and INSIG1 Bridge the Activation of TBK1 Kinase by Modifying the Adaptor STING. Immunity.

[32] Zhang J, Hu M-M, Wang Y-Y, Shu H-B (2012). TRIM32 Protein Modulates Type I Interferon Induction and Cellular Antiviral Response by Targeting MITA/STING Protein for K63-linked Ubiquitination. J Biol Chem.

[33] Ni G, Konno H, Barber GN. Ubiquitination of STING at lysine 224 controls IRF3 activation. Science Immunology 2017, 2(11). 10.1126/sciimmunol.aah7119 PMC5656267 28763789

[34] Tsuchida T, Zou J, Saitoh T, Kumar H, Abe T, Matsuura Y, Kawai T, Akira S (2010). The Ubiquitin Ligase TRIM56 Regulates Innate Immune Responses to Intracellular Double-Stranded DNA. Immunity.

[35] Wang Y, Lian Q, Yang B, Yan S, Zhou H, He L, Lin G, Lian Z, Jiang Z, Sun B. TRIM30 alpha Is a Negative-Feedback Regulator of the Intracellular DNA and DNA Virus-Triggered Response by Targeting STING. Plos Pathogens 2015, 11(6). 10.1371/journal.ppat.1005012 PMC4482643 26114947

[36] Zhong B, Zhang L, Lei C, Li Y, Mao A-P, Yang Y, Wang Y-Y, Zhang X-L, Shu H-B (2009). The Ubiquitin Ligase RNF5 Regulates Antiviral Responses by Mediating Degradation of the Adaptor Protein MITA. Immunity.

[37] Mukai K, Konno H, Akiba T, Uemura T, Waguri S, Kobayashi T, Barber GN, Arai H, Taguchi T. Activation of STING requires palmitoylation at the Golgi. Nature Communications 2016, 7. 10.1038/ncomms11932 PMC4919521 27324217

[38] Hansen AL, Buchan GJ, Ruehl M, Mukai K, Salvatore SR, Ogawa E, Andersen SD, Iversen MB, Thielke AL, Gunderstofte C (2018). Nitro-fatty acids are formed in response to virus infection and are potent inhibitors of STING palmitoylation and signaling. Proc Natl Acad Sci USA.

[39] Jia M, Qin D, Zhao C, Chai L, Yu Z, Wang W, Tong L, Lv L, Wang Y, Rehwinkel J, et al: Redox homeostasis maintained by GPX4 facilitates STING activation. Nature Immunology 2020. 10.1038/s41590-020-0699-0 32541831

[40] Hu M-M, Yang Q, Xie X-Q, Liao C-Y, Lin H, Liu T-T, Yin L, Shu H-B (2016). Sumoylation Promotes the Stability of the DNA Sensor cGAS and the Adaptor STING to Regulate the Kinetics of Response to DNA Virus. Immunity.

[41] Tao L, Lemoff A, Wang G, Zarek C, Lowe A, Yan N, Reese TA. Reactive oxygen species oxidize STING and suppress interferon production. eLife 2020, 9. 10.7554/eLife.57837 PMC7473769 32886065

[42] Blyth GAD, Connors L, Fodor C, Cobo ER. The Network of Colonic Host Defense Peptides as an Innate Immune Defense Against Enteropathogenic Bacteria. Frontiers in Immunology 2020, 11. 10.3389/fimmu.2020.00965 PMC7251035 32508838

[43] Woodward JJ, Iavarone AT, Portnoy DA (2010). c-di-AMP Secreted by Intracellular Listeria monocytogenes Activates a Host Type I Interferon Response. Science.

[44] Hansen K, Prabakaran T, Laustsen A, Jorgensen SE, Rahbaek SH, Jensen SB, Nielsen R, Leber JH, Decker T, Horan KA (2014). Listeria monocytogenes induces IFN beta expression through an IFI16-, cGAS- and STING-dependent pathway. Embo Journal.

[45] Zheng Z, Wei C, Guan K, Yuan Y, Zhang Y, Ma S, Cao Y, Wang F, Zhong H, He X (2016). Bacterial E3 Ubiquitin Ligase IpaH4.5 of Shigella flexneri Targets TBK1 To Dampen the Host Antibacterial Response. J Immunol.

[46] Dobbs N, Burnaevskiy N, Chen D, Gonugunta VK, Alto NM, Yan N (2015). STING Activation by Translocation from the ER Is Associated with Infection and Autoinflammatory Disease. Cell Host Microbe.

[47] Dong N, Zhu Y, Lu Q, Hu L, Zheng Y, Shao F (2012). Structurally Distinct Bacterial TBC-like GAPs Link Arf GTPase to Rab1 Inactivation to Counteract Host Defenses. Cell.

[48] Canesso MCC, Lemos L, Neves TC, Marim FM, Castro TBR, Veloso ES, Queiroz CP, Ahn J, Santiago HC, Martins FS (2018). The cytosolic sensor STING is required for intestinal homeostasis and control of inflammation. Mucosal Immunol.

[49] Park S-M, Omatsu T, Zhao Y, Yoshida N, Shah P, Zagani R, Reinecker H-C. T cell fate following Salmonella infection is determined by a STING-IRF1 signaling axis in mice. Communications Biology 2019, 2. 10.1038/s42003-019-0701-2 PMC6906324 31840109

[50] Levy MM, Evans LE, Rhodes A (2018). The Surviving Sepsis Campaign Bundle: 2018 Update. Crit Care Med.

[51] Oami T, Coopersmith CM (2019). A venomous relationship: Inflammation, the gut barrier and the STING pathway. Ebiomedicine.

[52] Hu Q, Ren H, Li G, Wang D, Zhou Q, Wu J, Zheng J, Huang J, Slade DA, Wu X (2019). STING-mediated intestinal barrier dysfunction contributes to lethal sepsis. Ebiomedicine.

[53] Zeng L, Kang R, Zhu S, Wang X, Cao L, Wang H, Billiar TR, Jiang J, Tang D. ALK is a therapeutic target for lethal sepsis. Science Translational Medicine 2017, 9(412). 10.1126/scitranslmed.aan5689 PMC5737927 29046432

[54] Samuels DC, Hulgan T, Fessel JP, Billings FT, Thompson JL, Chandrasekhar R, Girard TD (2019). Mitochondrial DNA Haplogroups and Delirium During Sepsis. Crit Care Med.

[55] Harrington JS, Huh J-W, Schenck EJ, Nakahira K, Siempos II, Choi AMK (2019). Circulating Mitochondrial DNA as Predictor of Mortality in Critically Ill Patients A Systematic Review of Clinical Studies. Chest.

[56] Zhang H, Zeng L, Xie M, Liu J, Zhou B, Wu R, Cao L, Kroemer G, Wang H, Billiar TR (2020). TMEM173 Drives Lethal Coagulation in Sepsis. Cell Host Microbe.

[57] Li N, Zhou H, Wu H, Wu Q, Duan M, Deng W, Tang Q. STING-IRF3 contributes to lipopolysaccharide-induced cardiac dysfunction, inflammation, apoptosis and pyroptosis by activating NLRP3. Redox Biology 2019, 24. 10.1016/j.redox.2019.101215 PMC6529775 31121492

[58] Zhang X, Bai XC, Chen ZJ (2020). Structures and Mechanisms in the cGAS-STING Innate Immunity Pathway. Immunity.

[59] Ahn J, Son S, Oliveira SC, Barber GN (2017). STING-Dependent Signaling Underlies IL-10 Controlled Inflammatory Colitis. Cell Reports.

[60] Martin GR, Blomquist CM, Henare KL, Jirik FR. Stimulator of interferon genes (STING) activation exacerbates experimental colitis in mice. Scientific Reports 2019, 9. 10.1038/s41598-019-50656-5 PMC6776661 31582793

[61] Chang EY, Guo B, Doyle SE, Cheng G (2007). Cutting edge: Involvement of the type IIFN production and signaling pathway in lipopolysaccharide-induced IL-10 production. J Immunol.

[62] Aden K, Tran F, Ito G, Sheibani-Tezerji R, Lipinski S, Kuiper JW, Tschurtschenthaler M, Saveljeva S, Bhattacharyya J, Haesler R (2018). ATG16L1 orchestrates interleukin-22 signaling in the intestinal epithelium via cGAS-STING. J Exp Med.

[63] Ma C, Yang D, Wang B, Wu C, Wu Y, Li S, Liu X, Lassen K, Dai L, Yang S. Gasdermin D in macrophages restrains colitis by controlling cGAS-mediated inflammation. Science Advances 2020, 6(21). 10.1126/sciadv.aaz6717 PMC7314554 32671214

[64] Ahn J, Konno H, Barber GN (2015). Diverse roles of STING-dependent signaling on the development of cancer. Oncogene.

[65] Irrazabal T, Belcheva A, Girardin SE, Martin A, Philpott DJ (2014). The Multifaceted Role of the Intestinal Microbiota in Colon Cancer. Mol Cell.

[66] Oke S, Martin A (2017). Insights into the role of the intestinal microbiota in colon cancer. Therapeutic Advances in Gastroenterology.

[67] Song S, Peng P, Tang Z, Zhao J, Wu W, Li H, Shao M, Li L, Yang C, Duan F, et al: Decreased expression of STING predicts poor prognosis in patients with gastric cancer. Scientific Reports 2017, 7. 10.1038/srep39858 PMC5296877 28176788

[68] Kuse N, Sun X, Akahoshi T, Lissina A, Yamamoto T, Appay V, Takiguchi M (2019). Priming of HIV-1-specific CD8(+) T cells with strong functional properties from naive T cells. Ebiomedicine.

[69] Barber GN (2015). STING: infection, inflammation and cancer. Nat Rev Immunol.

[70] ChonL HJ, Kim H, Noh JH, Yang H, Lee WS, Kong SJ, Lee SJ, Lee YS, Kim WR, Kim JH (2019). STING signaling is a potential immunotherapeutic target in colorectal cancer. J Cancer.

[71] Woo S-R, Fuertes MB, Corrales L, Spranger S, Furdyna MJ, Leung MYK, Duggan R, Wang Y, Barber GN, Fitzgerald KA (2014). STING-Dependent Cytosolic DNA Sensing Mediates Innate Immune Recognition of Immunogenic Tumors. Immunity.

[72] McWhirter SM, Jefferies CA (2020). Nucleic Acid Sensors as Therapeutic Targets for Human Disease. Immunity.

[73] Corrales L, Glickman LH, McWhirter SM, Kanne DB, Sivick KE, Katibah GE, Woo S-R, Lemmens E, Banda T, Leong JJ (2015). Direct Activation of STING in the Tumor Microenvironment Leads to Potent and Systemic Tumor Regression and Immunity. Cell Reports.

[74] Fu J, Kanne DB, Leong M, Glickman LH, McWhirter SM, Lemmens E, Mechette K, Leong JJ, Lauer P, Liu W, et al: STING agonist formulated cancer vaccines can cure established tumors resistant to PD-1 blockade. Science Translational Medicine 2015, 7(283). 10.1126/scitranslmed.aaa4306 PMC4504692 25877890

[75] Chin EN, Yu C, Vartabedian VF, Jia Y, Kumar M, Gamo AM, Vernier W, Ali SH, Kissai M, Lazar DC (2020). Antitumor activity of a systemic STING-activating non-nucleotide cGAMP mimetic. Science.

[76] Lemos H, Mohamed E, Huang L, Ou R, Pacholczyk G, Arbab AS, Munn D, Mellor AL (2016). STING Promotes the Growth of Tumors Characterized by Low Antigenicity via IDO Activation. Cancer research.

[77] Fu J, Kanne DB, Leong M, Glickman LH, McWhirter SM, Lemmens E, Mechette K, Leong JJ, Lauer P, Liu W (2015). STING agonist formulated cancer vaccines can cure established tumors resistant to PD-1 blockade. Sci Transl Med.

[78] Zheng J, Mo J, Zhu T, Zhuo W, Yi Y, Hu S, Yin J, Zhang W, Zhou H, Liu Z (2020). Comprehensive elaboration of the cGAS-STING signaling axis in cancer development and immunotherapy. Mol Cancer.

[79] Bakhoum SF, Ngo B, Laughney AM, Cavallo JA, Murphy CJ, Ly P, Shah P, Sriram RK, Watkins TBK, Taunk NK (2018). Chromosomal instability drives metastasis through a cytosolic DNA response. Nature.

[80] Chen Q, Boire A, Jin X, Valiente M, Er EE, Lopez-Soto A, Jacob L, Patwa R, Shah H, Xu K (2016). Carcinoma-astrocyte gap junctions promote brain metastasis by cGAMP transfer. Nature.

[81] Gulen MF, Koch U, Haag SM, Schuler F, Apetoh L, Villunger A, Radtke F, Ablasser A (2017). Signalling strength determines proapoptotic functions of STING. Nat Commun.

[82] Hu QY, Wu J, Ren YH, Wu XW, Gao L, Wang GF, Gu GS, Ren HJ, Hong ZW, Slade DA (2020). Degree of STING activation is associated with disease outcomes. Gut.

[83] Zmora N, Suez J, Elinav E (2019). You are what you eat: diet, health and the gut microbiota. Nature reviews Gastroenterology hepatology.

[84] Wu J-J, Zhao L, Hu H-G, Li W-H, Li Y-M (2020). Agonists and inhibitors of the STING pathway: Potential agents for immunotherapy. Med Res Rev.

[85] Vermaelen K. Vaccine Strategies to Improve Anti-cancer Cellular Immune Responses. Frontiers in Immunology 2019, 10. 10.3389/fimmu.2019.00008 PMC6349827 30723469

[86] Koshy ST, Cheung AS, Gu L, Graveline AR, Mooney DJ: Liposomal Delivery Enhances Immune Activation by STING Agonists for Cancer Immunotherapy. Advanced Biosystems 2017, 1(1–2). 10.1002/adbi.201600013 PMC6152940 30258983

[87] Cheng N, Watkins-Schulz R, Junkins RD, David CN, Johnson BM, Montgomery SA, Peine KJ, Darr DB, Yuan H, McKinnon KP (2018). A nanoparticle-incorporated STING activator enhances antitumor immunity in PD-L1-insensitive models of triple-negative breast cancer. JCI Insight.

[88] Luo M, Wang H, Wang Z, Cai H, Lu Z, Li Y, Du M, Huang G, Wang C, Chen X (2017). A STING-activating nanovaccine for cancer immunotherapy. Nat Nanotechnol.

[89] Zhou L, Hou B, Wang D, Sun F, Song R, Shao Q, Wang H, Yu H, Li Y (2020). Engineering Polymeric Prodrug Nanoplatform for Vaccination Immunotherapy of Cancer. Nano Lett.

[90] Lu X, Miao L, Gao W, Chen Z, McHugh KJ, Sun Y, Tochka Z, Tomasic S, Sadtler K, Hyacinthe A, et al: Engineered PLGA microparticles for long-term, pulsatile release of STING agonist for cancer immunotherapy. Science translational medicine 2020, 12(556). 10.1126/scitranslmed.aaz6606 PMC9019818 32801144

[91] Collier MA, Junkins RD, Gallovic MD, Johnson BM, Johnson MM, Macintyre AN, Sempowski GD, Bachelder EM, Ting JPY, Ainslie KM (2018). Acetalated Dextran Microparticles for Codelivery of STING and TLR7/8 Agonists. Mol Pharm.

[92] Junkins RD, Gallovic MD, Johnson BM, Collier MA, Watkins-Schulz R, Cheng N, David CN, McGee CE, Sempowski GD, Shterev I (2018). A robust microparticle platform for a STING-targeted adjuvant that enhances both humoral and cellular immunity during vaccination. J Controlled Release.

[93] Leach DG, Dharmaraj N, Piotrowski SL, Lopez-Silva TL, Lei YL, Sikora AG, Young S, Hartgerink JD (2018). STINGel: Controlled release of a cyclic dinucleotide for enhanced cancer immunotherapy. Biomaterials.

[94] Wang F, Su H, Xu D, Dai W, Zhang W, Wang Z, Anderson CF, Zheng M, Oh R, Wan F, et al: Tumour sensitization via the extended intratumoural release of a STING agonist and camptothecin from a self-assembled hydrogel. Nature biomedical engineering 2020. 10.1038/s41551-020-0597-7 PMC8848303 32778697

[95] Snell LM, McGaha TL, Brooks DG (2017). Type I Interferon in Chronic Virus Infection and Cancer. Trends Immunol.

[96] Kretschmer S, Lee-Kirsch MA (2017). Type I interferon-mediated autoinflammation and autoimmunity. Curr Opin Immunol.

[97] Zitvogel L, Galluzzi L, Kepp O, Smyth MJ, Kroemer G (2015). Type I interferons in anticancer immunity. Nature reviews Immunology.

[98] Budhwani M, Mazzieri R, Dolcetti R (2018). Plasticity of Type I Interferon-Mediated Responses in Cancer Therapy: From Anti-tumor Immunity to Resistance. Frontiers in oncology.

